# Diversification of polyphosphate end-labeling via bridging molecules

**DOI:** 10.1371/journal.pone.0237849

**Published:** 2020-08-21

**Authors:** Catherine J. Baker, Stephanie A. Smith, James H. Morrissey

**Affiliations:** Department of Biological Chemistry, University of Michigan Medical School, Ann Arbor, Michigan, United States of America; Stanford University, UNITED STATES

## Abstract

Investigation of the biological roles of inorganic polyphosphate has been facilitated by our previous development of a carbodiimide-based method for covalently coupling primary amine-containing molecules to the terminal phosphates of polyphosphate. We now extend that approach by optimizing the reaction conditions and using readily available “bridging molecules” containing a primary amine and an additional reactive moiety, including another primary amine, a thiol or a click chemistry reagent such as dibenzocyclooctyne. This two-step labeling method is used to covalently attach commercially available derivatives of biotin, peptide epitope tags, and fluorescent dyes to the terminal phosphates of polyphosphate. Additionally, we report three facile methods for purifying conjugated polyphosphate from excess reactants.

## Introduction

Polyphosphate (polyP) is a linear polymer of inorganic orthophosphates that is widespread in biology [1]. Recent years have seen rapidly increasing interest in understanding the biological roles of this polymer in both lower organisms and mammals [2–5]. However, progress in this area has been hampered by a dearth of methods and tools for manipulating and analyzing polyP. We previously reported a method for covalently coupling the terminal phosphates of polyP to primary amine-containing molecules via phosphoramidate linkages, in a reaction mediated by the water-soluble carbodiimide, *N*-(3-dimethylaminopropyl)-*N′*-ethylcarbodiimide (EDAC) [6]. There are limitations to this method, however. The desired label must be water-soluble and contain a non-aryl primary amine, limiting the type of labeling molecules that can readily be coupled to polyP. Furthermore, if the label also contains reactive groups such as carboxylates, EDAC is likely to promote unwanted polymerization. And more recently, we have found that some reaction conditions reported in our previous study [6] can promote polyP degradation. Since biological origins of polyP affect its length and size distribution [7], preserving those characteristics can aid in our understanding of a given sample’s biological functions. In the present study, we sought to expand the possibilities for covalently end-labeling polyP to take advantage of the large variety of coupling chemistries available for derivatizing proteins. We now report optimized reaction conditions for attaching bridging molecules in the form of peptides or small molecules to the terminal phosphates of polyP, thereby permitting the use of a wider variety of commercially available labels. We also report coupling conditions that better preserve the polymer length of polyP, and the use of three facile methods for purifying the end-labeled polyP.

## Materials and methods

### Materials

**Fig 1:**
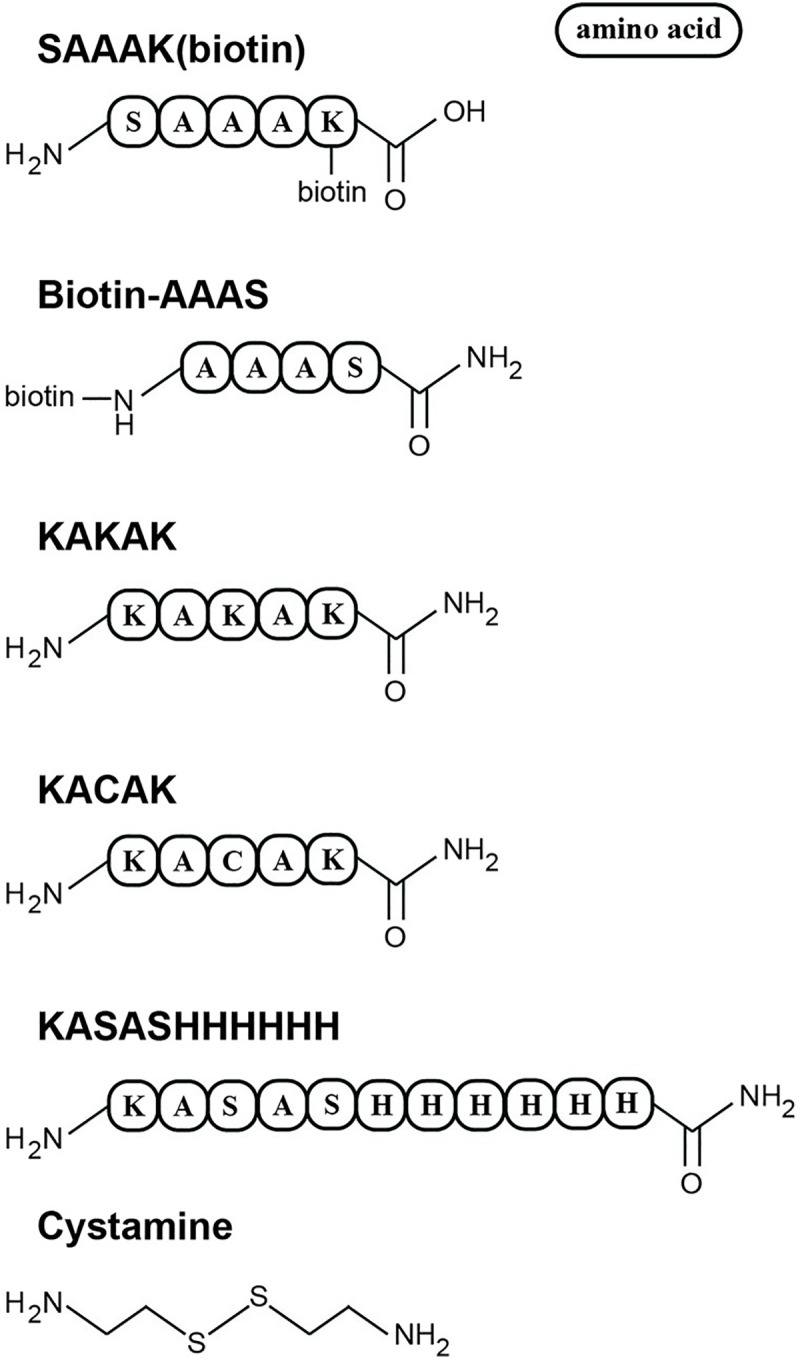
Custom peptides and cystamine used as bridging molecules with polyP. Peptides were synthesized either with the label already attached (such as SAAAK (biotin) or biotin-AAAS) or with reactive groups that allow the use of amine- or cysteine-reactive chemistries to attach labels in secondary reactions that use the peptide or cystamine as a “bridging molecule”.

EDAC, 4-(*N*-morpholino)butanesulfonic acid (MOBS), 3-(N-morpholino)propanesulfonic acid (MOPS), 3-(cyclohexylamino)-1-propanesulfonic acid (CAPS), fluorescamine, toluidine blue, Ficoll 400, cystamine dihydrochloride, and water-insoluble, high-molecular-weight polyP were from Millipore Sigma. Zeba spin desalting columns (7,000 molecular weight cutoff), EZ-Link amine-PEG_2_-biotin, sulfo-NHS-LC-biotin, EZ-Link maleimide-PEG_2_-biotin, DyLight 488 NHS ester, DyLight 488 maleimide, Nunc Streptavidin Immobilizer 8-well strip plates, Pierce Fluorescence Biotin Quantitation Kit and tris(2-carboxyethyl)phosphine hydrochloride (TCEP) were from ThermoFisher. The following custom oligopeptides (Fig 1) were from the following suppliers: KASASHHHHHH, KACAK, and KAKAK from GenScript; and SAAAK(biotin) (with biotin covalently attached to the ε-amino group of lysine), biotin-AAAS (with biotin covalently attached to the N-terminus) and KAKAK from ThermoFisher. Biotin-PEG_3_-azide and dibenzocyclooctyne (DBCO)-PEG_4_-amine conjugates were from Jena Bioscience, Sar-Pro-Arg-*p-*nitroanilide was from Bachem, bovine thrombin was from BioPharm, and Ni-NTA spin kits were from Qiagen. All other reagents were from Fisher Scientific. Polyacrylamide gel electrophoresis was performed using Mini-Protean 4–20% TBE gels from Bio-Rad. FPLC chromatography was performed on an Äkta Pure system using Superdex 30 Increase 10/300 GL columns (GE Healthcare).

## Methods

Heterogenous long-chain polyP (polyP_1000_), with a modal polymer length of 1000 phosphates and a range of 200 to 1300 phosphates, was solubilized from high-molecular-weight polyP using base hydrolysis followed by isopropanol precipitation, as described [8]. A more homogeneous sample of long-chain polyP (polyP_narrow_), with a length range of 1000–1300 phosphates, was isolated using preparative polyacrylamide gel electrophoresis as described [7]. Heterogeneous short-chain polyP (polyP_75_), with a modal polymer length of 75 phosphates and a range of 20–280 phosphates, was prepared using differential acetone precipitation of P70 (a kind gift from Dr. Thomas Staffel, BK Giulini GmbH). Briefly, to a stirred solution of 500 mM P70 in 100 mM NaCl, acetone was gradually added to a final concentration of 12.2% v/v. The precipitated polyP was collected by centrifugation for 20 min at 13,000 × *g*, dried under nitrogen gas and dissolved in a solution of 20 mM Hepes-NaOH buffer (pH 7.4).

PolyP concentrations were quantified using a malachite green assay after complete acid hydrolysis, as previously described [9]. Note that since polyP polymers are polydisperse, all polyP concentrations throughout this report are given in terms of the concentration of phosphate monomer.

### EDAC-mediated phosphoramidate linkages

Unless otherwise stated, reaction mixtures contained polyP at a concentration equivalent to 100 μM ends; in terms of phosphate monomer concentration, this was 50 mM for polyP_1000_ and polyP_narrow_, or 3.75 mM for polyP_75_. The other typical reaction components were 150 mM EDAC (from a freshly made stock solution), 8 mM amine-containing label of interest, and, when included, 100 mM buffer. EDAC was added last, and the reagents were mixed by vortexing and incubated at 37° or 60°C (typically, for 1 h), then chilled on ice prior to purification. Unreacted label was removed using either a desalting spin column, acetone precipitation, or FPLC-based size-exclusion chromatography (SEC) as described below. Unless otherwise noted, samples were purified into a solution of 20 mM Hepes-NaOH buffer (pH 7.4) containing 5 or 10 mM EDTA (termed HE5 or HE10 buffer, respectively).

### Optimizing reaction pH

1.5 mL of polyP/EDAC reaction mixture was prepared in the absence of buffer with amine-PEG_2_-biotin as the label, then evenly divided into 7 samples to which either HCl or NaOH was added to create a range of pH values from 6.0 to 9.5. Each mixture was incubated at 37°C for 1 h and chilled on ice, from which polyP was recovered by acetone precipitation. After quantitation by malachite green assay, each sample was diluted to 4 μM phosphate, and 100 μL was used in a modified thrombin-binding assay [7] (described below) to assess labeling efficiency.

### Effect of buffer on labeling efficiency

1.5 mL of polyP/EDAC reaction mixture was prepared in the absence of buffer with amine-PEG_2_-biotin as the label, then aliquoted into 3 samples which were brought to pH 8.0 by either adding concentrated pH 8.0 MOBS-NaOH or MOPS-NaOH buffers (100 mM final concentration), or by adjusting the pH of the unbuffered solution to 8.0 with HCl. Samples were incubated at 37°C for 1 h, stopped on ice, purified and evaluated by thrombin-binding assay.

### Acetone precipitation

CAPS buffer pH 9.8 (35 mM final) and NaCl (270 mM final) were added to samples, followed by two reaction volumes of acetone, mixing and centrifugation at 5000 × *g* for 10 min. Pellets were collected and washed twice with 3× the initial reaction volume of acetone, after which the precipitates were dried and reconstituted in desired buffer.

### Spin column-based SEC and biotin quantification

Zeba spin desalting columns were used according to the manufacturer’s instructions. To determine recovery efficiency using polyP_1000_, 300 μL of reaction mixture was added to a pre-equilibrated (as described for each experiment) 2 mL spin column and centrifuged at 1000 × *g* for 2 min. Aliquots from before and after the spin-column purification were acid-hydrolyzed and phosphate concentrations quantified by malachite green [9]. Biotin concentrations were measured using the Pierce Fluorescence Biotin Quantification kit according to the manufacturer’s instructions.

To determine if the shorter polyP_75_ preparation can also be recovered efficiently from these spin columns, a 4.23 mM polyP_75_ solution was divided into 4 aliquots. Each aliquot was separated on a 2-mL spin desalting column that had been pre-equilibrated with 100 mM MOPS pH 8.0 following the manufacturer’s instructions. The flow-through was collected and ensured that the volume remained consistent. Then all samples were hydrolyzed and polyP content quantified by malachite green assay.

### FPLC-based SEC

An Äkta Pure system was used to resolve polyP on a Superdex 30 Increase 10/300 GL column, typically running at 0.8 mL/min in 20 mM Hepes-NaOH buffer (pH 7.4) and collecting 0.75 mL fractions. Fractions were tested for polyP by adding 50 μL of each fraction to microplate wells containing 100 μL of 3.8 mg/L toluidine blue in 0.025% acetic acid, and measuring the A_540_/A_640_ ratio to identify polyP-containing fractions. Cystamine content was monitored by fluorescence (excitation: 365 nm; emission: 470 nm) in 96-well black polypropylene microplates after mixing 50 μL of fractions with 20 μL of a solution of 1 mg/mL fluorescamine in acetone.

### Secondary reactions of derivatized polyP

#### Primary amine-reactive

After the initial EDAC-mediated coupling of polyP with an amine-containing bridging molecule that had one or more additional primary amines (e.g., KAKAK or cystamine), samples were purified into 100 mM pH 8.0 buffer (either MOPS or MOBS). Secondary labels (8 mM sulfo-NHS-LC-biotin or NHS-DyLight488) were added to the purified, amine-derivatized polyP, and mixtures were rotated at room temperature for 1.5 h. Samples were then purified into HE5 buffer for storage, analysis and future use.

#### Thiol-reactive

PolyP that had been coupled to cystamine or KACAK using EDAC, then purified, was mixed with 5 mM TCEP in HE10 buffer and rotated at room temperature for 1.5 h. TCEP was then removed using spin desalting columns equilibrated with HE10 buffer. The desired thiol-reactive label (8 mM maleimide-PEG_2_-biotin or DyLight-488-maleimide) was immediately added to the reduced sample and the mixture was rotated at room temperature for 1.5 h, then purified into HE10 buffer. We have made a detailed protocol available online for EDAC-mediated coupling of cystamine to polyP_1000_ followed by TCEP reduction and end-labeling with maleimide-PEG_2_-biotin: http://dx.doi.org/10.17504/protocols.io.7z3hp8n.

### Copper-free click chemistry

5 mM biotin-PEG_3_-azide in HE5 buffer was added to polyP that had previously been coupled to the bridging molecule, DBCO-PEG_4_-amine, using EDAC. Samples were rotated at room temperature for 4 h, then purified on a spin-desalting column equilibrated with HE5 buffer.

### Gel electrophoresis visualization of polyP

PolyP_narrow_ samples were diluted to 1–10 mM (in terms of phosphate monomer) and resolved on 4–20% gradient TBE gels at 150 V until the bromophenol blue neared the bottom. For fluorescent labels, gels were imaged first using UV light (illumination at 302 nm, with a 595 nm emission filter), and then stained for polyP using 0.05% toluidine blue in 25% methanol/5% glycerol, followed by destaining in 25% methanol/5% glycerol and imaged using white light.

### Determination of biotinylation efficiency by thrombin-binding assay

A microplate-based thrombin-binding assay for immobilized polyP was performed as described [10]. Briefly, varying concentrations of biotinylated polyP (based on phosphate monomer concentration) in 50 mM Tris-HCl buffer pH 7.4, 1% bovine serum albumin, 5 mM EDTA, 0.05% Tween 20 were incubated in streptavidin-coated 96-well microplates for 3 h at room temperature to capture biotin-polyP. After washing, wells were incubated for 1 h with 40 nM bovine thrombin in 20 mM Hepes-NaOH pH 7.4, 5mM EDTA, 50 mM NaCl, 0.1% bovine serum albumin, and 0.05% Tween 20. After washing again, the bound thrombin was detected spectrophotometrically (A_405_) by rate cleavage of 0.4 mM Sar-Pro-Arg-*p-*nitroanilide substrate. For each plot, rates of chromogenic substrate cleavage were normalized to the highest value obtained (and plotted as % maximal signal). The concept for this assay is that the higher the percentage of polyP that is biotinylated, the more polyP is captured on the microplate and therefore the more thrombin that is subsequently bound. The high-throughput nature of this assay makes it particularly suitable for this investigation. Each analysis requires only 1–10 μg of labeled material and the relative labeling efficiency is easily compared between experiments.

### Purification and quantification of His-tagged polyP

PolyP was coupled to the custom peptide KASASHHHHHH (generating His-tag-polyP), then purified using a desalting column pre-equilibrated with 100 mM MOPS-NaOH buffer (pH 8.0). Then, 600 μL of a solution of His-tag-polyP in 100 mM MOPS-NaOH buffer (pH 8.0), 300 mM NaCl, 3 mM imidazole was applied to a Ni-NTA spin column that had previously been equilibrated with 80 mM MOPS-NaOH buffer (pH 8.0), 300 mM NaCl, 10 mM imidazole. After centrifugation at 270 × *g* for 5 min, the column was washed 4 times by centrifugation (900 × *g* for 2 min) with 600 μL wash buffer (80 mM MOPS-NaOH buffer (pH 8.0), 300 mM NaCl, 20 mM imidazole). PolyP was eluted in two fractions by adding 300 μL elution buffer (80 mM MOPS-NaOH buffer pH 8.0, 300 mM NaCl, 500 mM imidazole) and centrifuging at 900 × *g* for 2 min. These elutions were pooled and buffer-exchanged using the spin-desalting columns into 100 mM MOPS-NaOH buffer (pH 8.0), 300 mM NaCl, 3 mM imidazole before being added to another equilibrated Ni-NTA column. The entire Ni-NTA purification procedure was repeated for 2 washes and 3 elutions, after which the phosphate concentration of each fraction was quantified by acid hydrolysis/malachite green.

## Results and discussion

### Optimization of phosphoramidation reaction conditions

**Fig 2:**
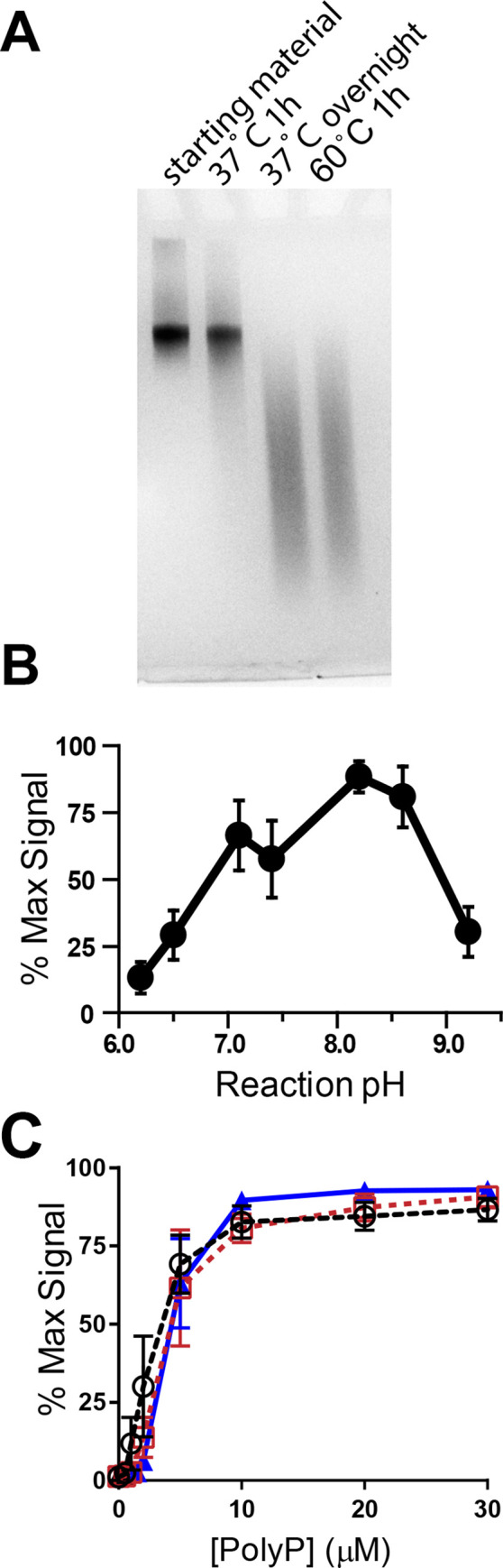
Effect of time, temperature and pH on coupling of polyP to amine-PEG_2_-biotin. **A**) PolyP_narrow_ was reacted with EDAC and amine-PEG_2_-biotin at unbuffered pH 8 at 37°C for 1 h or overnight, or at 60°C for 1 h, then resolved by PAGE. The fidelity of the size distribution was preserved in the 37°C 1 h reaction, but serious degradation was observed in the other reaction conditions. **B**) To determine the optimal reaction pH, polyP_1000_ was reacted with amine-PEG_2_-biotin and EDAC for 1 h at 37°C at a variety of unbuffered pH solutions. After purification, each was evaluated by thrombin-binding assay and the results were normalized to the highest value obtained. **C**) To determine if the inclusion of buffers inhibited the EDAC-mediated coupling reactions, polyP_1000_ was reacted with amine-PEG_2_-biotin and EDAC for 1 h at 37°C in a reaction mixture that included pH 8.0 MOBS-NaOH (□) or pH 8.0 MOPS-NaOH (▲) buffers, or by adjusting the pH of the unbuffered solution to 8.0 with HCl (○). After purification, the coupling efficiency of each preparation was evaluated by thrombin-binding assay, with the results normalized to the highest value obtained.

Anecdotally, we noted variable shortening of polyP chains when employing our previously published conditions to couple primary amines to polyP [6] (although this can be difficult to discern when labeling polyP preparations with broad size distributions). To examine this directly, we incubated a narrowly size-fractionated preparation of long-chain polyP (1000–1300 phosphates long, termed polyP_narrow_) in the presence of EDAC and amine-PEG_2_-biotin but without buffer, at 37°C or 60°C. Resolving the purified polyP on PAGE revealed that reactions performed at 37°C for 1 h preserved the original polyP size distribution, while overnight reactions at 37°C, or 1-hr reactions at 60°C, resulted in substantial shortening of polyP chains (Fig 2A). Therefore, when preserving polyP length is important, reacting with EDAC for 1 hr at 37°C is recommended.

In our initial publication of EDAC-mediated labeling of polyP, we reported that including Ca^2+^ could increase the apparent labeling efficiency [6]. However, we have subsequently found that free Ca^2+^ can promote polyP degradation [11]. It seems likely, therefore, that including Ca^2+^ in EDAC-mediated labeling reactions increases labeling efficiency by promoting chain cleavage and thus increasing the number of polyP ends that can react with primary amines. Accordingly, Ca^2+^ should be avoided when reacting polyP, in order to preserve the polyP polymer length.

We also analyzed the effect of reaction pH on coupling efficiency by reacting polyP_1000_ with amine-PEG_2_-biotin and EDAC at a variety of unbuffered pH values. Extent of labeling was quantified by thrombin-binding assay (Fig 2B). Peak labeling efficiency occurred at pH 8 to 8.5. Inclusion of pH 8.0 buffer (100 mM MOPS-NaOH or MOBS-NaOH) in reaction mixtures did not alter coupling efficiency relative to unbuffered mixtures adjusted to 8.0 (Fig 2C). When selecting a buffer, it is important to avoid moieties such as primary amines or carboxylates that can react in the presence of EDAC, and MOPS and MOBS lack such reactive groups. Since MOPS is less expensive than MOBS, we therefore chose to routinely use pH 8.0 MOPS-NaOH buffer when coupling polyP to primary amines.

### Labeling polyP with peptides

To examine the use of peptides as bridging molecules for polyP end-labeling, five oligopeptides were synthesized that contained at least one free primary amine or hydroxyl group (Table 1).

**Table 1:** Amino acid sequences of oligopeptides and their modifications.

Name	N-terminus	Amino acid sequence	Side-chain modification	C-terminus
SAAAK(biotin)	NH_2_–	SAAAK	Lysine biotinylation	–COOH
Biotin-AAAS	Biotin-NH_2_–	AAAS	None	–CONH_2_
KAKAK	NH_2_–	KAKAK	None	–CONH_2_
KACAK	NH_2_–	KACAK	None	–CONH_2_
KASASHHHHHH	NH_2_–	KASASHHHHHH	None	–CONH_2_

**Fig 3:**
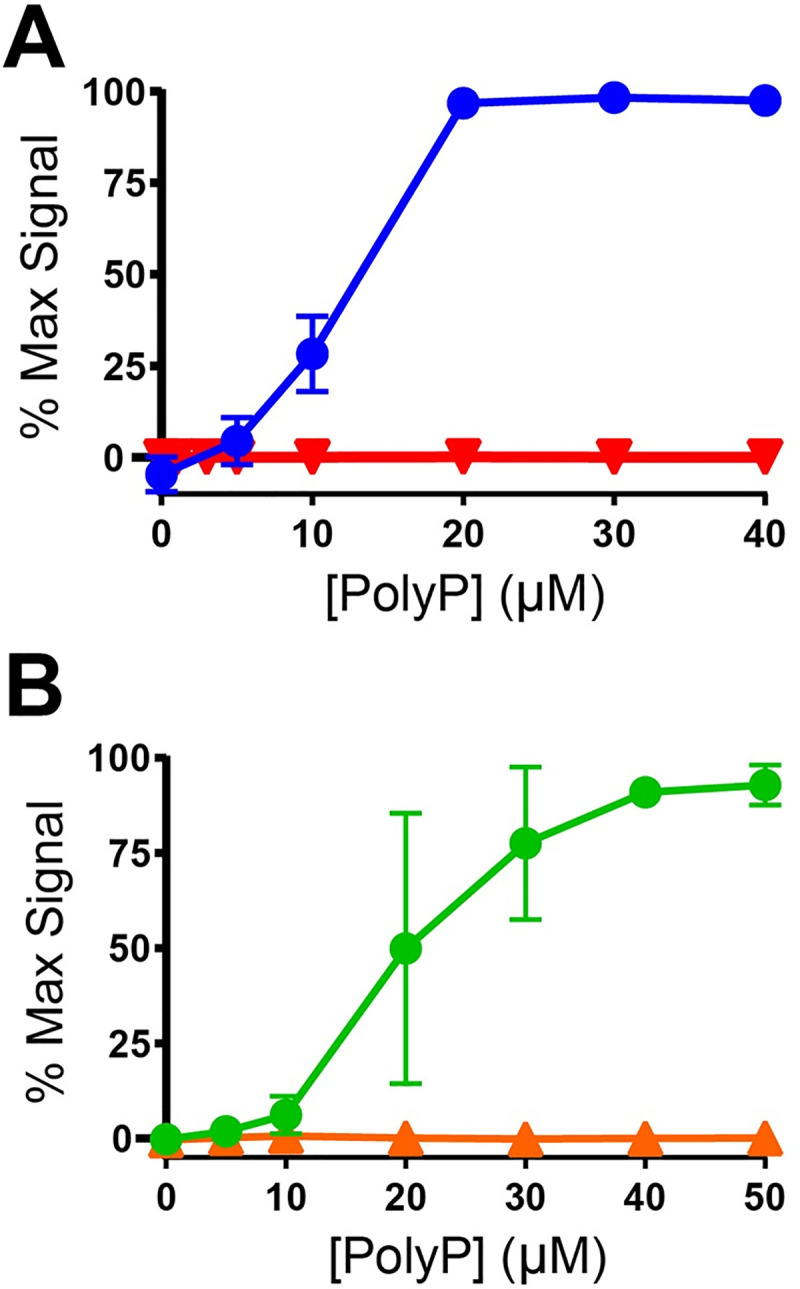
Peptides can be used to covalently biotinylate polyP. **A**) PolyP_1000_ was reacted with EDAC and a biotinylated peptide, either SAAAK(biotin) (●), or biotin-AAAS (▼), and then purified. Degree of polyP biotinylation was assessed by the microplate-based thrombin-binding assay. **B**) PolyP_1000_ was reacted with EDAC in the presence of the peptide, KAKAK (●) or in the absence of peptide (▲). Following purification, polyP preparations were incubated with sulfo-NHS-LC-biotin and repurified. Extent of biotinylation was assessed by the thrombin-binding assay.

Two of the peptides were synthesized with covalently attached biotin moieties: SAAAK(biotin) with a biotin attached to an internal lysine residue, and biotin-AAAS with a biotin attached to the N-terminus. We reacted each with polyP_1000_ and EDAC and tested the degree of biotin labeling using the thrombin-binding assay. SAAAK(biotin) had a free amino-terminus and we found that it coupled to polyP under the reaction conditions tested (Fig 3A). Biotin-AAAS had no free primary amines but had a free serine hydroxyl group. We attempted to couple this peptide to polyP, because we had previously shown that EDAC can promote the formation of phosphoester bonds between alcohols and polyP [12]. The thrombin-binding assay showed no evidence of biotinylation of polyP using this approach, however (Fig 3A). In our previous report of coupling hydroxyl-containing compounds to polyP, we found it necessary to employ very high concentrations of alcohols (0.2 to 6.4 M). It seems likely that the lack of detectable coupling of polyP to the biotin-AAAS peptide was due to the relatively low concentration of this peptide in the coupling reaction (8 mM). Higher peptide concentrations were not feasible owing to solubility limits and costs of synthetic peptides.

### Peptides and cystamine as bridging molecules for polyP labeling

We tested the idea that a peptide with two or more free primary amines could be coupled to polyP via EDAC reaction, which would then serve as a bridging molecule to allow the use of amine-reactive probes to covalently derivatize polyP. Accordingly, KAKAK, a peptide with 4 primary amines including the N-terminus, was reacted with polyP_1000_ via the standard EDAC reaction. After purification, the KAKAK-coupled polyP was incubated with the amine-reactive label, sulfo-NHS-LC biotin, and purified again. The thrombin-binding assay demonstrated successful attachment of sulfo-NHS-LC biotin to KAKAK-coupled polyP_1000_, while polyP_1000_ that had not been reacted with KAKAK showed no evidence of biotinylation (Fig 3B). Despite the fact that the peptides contain multiple primary amines, the effective secondary labeling indicates that polymer networks were not formed by the EDAC reaction. This may be due to the high ratio of label to polyP ends (80:1) and low ionic strength of the reaction mixture, causing the polymers to repel one another.

**Fig 4:**
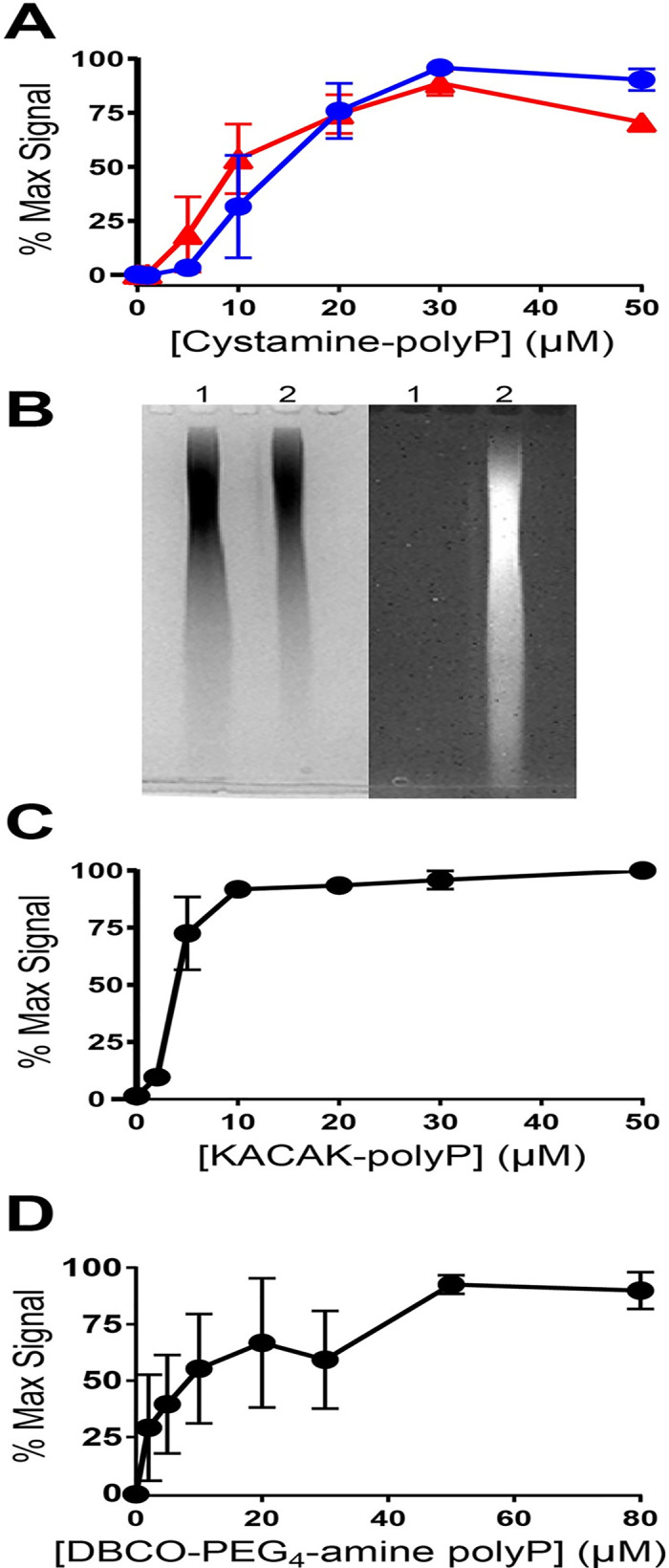
Coupling chemistries for polyP attached to three different bridging linkers. **A**) Biotinylation of cystamine-coupled polyP. After EDAC-mediated coupling of cystamine to polyP_1000_, the resulting conjugate was reacted with sulfo-NHS-LC-biotin (●), or was reduced with TCEP and reacted with maleimide-PEG_2_-biotin (▲). Extent of biotinylation was evaluated using the thrombin-binding assay. **B**) Fluorescent labeling of cystamine-coupled polyP, resolved on PAGE. The image on the left is the gel following staining with toluidine blue, photographed with white light, while the image on the right is the same gel before toluidine staining, photographed under UV illumination. Lane 1, starting polyP_1000_ preparation. Lane 2, after coupling cystamine to polyP_1000_, the resulting conjugate was reduced with TCEP and reacted with maleimide-DyLight488, then purified again. **C**) Biotinylation of peptide-coupled polyP via sulfhydryl labeling. After EDAC-mediated coupling of the peptide, KACAK, to polyP, the resulting conjugate was reduced with TCEP and reacted with maleimide-PEG_2_-biotin. Extent of biotinylation was evaluated with the thrombin-binding assay. **D**) Biotinylation of polyP using copper-free click chemistry. After EDAC-mediated coupling of DBCO-PEG_4_-amine to polyP_1000_, the resulting conjugate was reacted with biotin-PEG_3_-azide and purified. Extent of biotinylation was evaluated with the thrombin-binding assay.

We next investigated the use of cystamine as a cheaper and more versatile bridging molecule for attaching either amine- or thiol-reactive materials to the terminal phosphates of polyP. (Previously, cystamine was successfully employed to create polyP-functionalized nanoparticles [13].) We first coupled cystamine to polyP_1000_ using EDAC, and after purification, either reacted the cystamine-coupled polyP directly with sulfo-NHS-LC-biotin, or reduced the cystamine-coupled polyP with TCEP and reacted it with maleimide-PEG_2_-biotin. The thrombin-binding assay showed efficient biotinylation of polyP using either approach (Fig 4A). One potential advantage of using an amine-reactive probe such as sulfo-NHS-LC-biotin to couple biotin to cystamine-derivatized polyP is that the biotin moiety could subsequently be removed from polyP by a reducing agent such as dithiothreitol.

We also incubated the sulfhydryl-reactive dye, maleimide-Dylight488, with cystamine-coupled polyP_1000_ (after reduction with TCEP) and confirmed fluorescent labeling of polyP (Fig 4B). (And we note that this PAGE profile indicated no apparent diminution in the polyP length during the labeling procedure.) As an alternative way of introducing thiols onto the ends of polyP, we used EDAC to couple the cysteine-containing peptide, KACAK, to polyP_1000_. After reduction with TCEP and incubation with maleimide-PEG_2_-biotin, we found robust evidence of polyP biotinylation (Fig 4C).

We also examined the feasibility of attaching click chemistry reagents to polyP by first coupling DBCO-PEG_4_-amine to polyP_1000_ in the presence of EDAC. After purification, the DBCO-derivatized polyP was incubated with biotin-PEG_3_-azide and purified again. The thrombin-binding assay showed efficient biotinylation of polyP using this approach (Fig 4D). Compatibility with copper-free click chemistry reactions therefore further widens the approaches for end-labeling polyP.

Finally, we explored the feasibility of attaching a peptidyl epitope tag to polyP. Accordingly, KASASHHHHHH was reacted with polyP_1000_ and EDAC to add the His-tag functionality to the polymer. After reaction and purification using spin-desalting columns, the His-tagged polyP was purified twice (sequentially) using a Ni-NTA spin column. 74% of the chromatographed polyP was eluted the second time with imidazole, confirming the attachment of the affinity tag to the polyP. Unfortunately, this accounted for only 15% of the initial polyP. This peptide proved difficult at every stage: the vendor struggled to synthesize a peptide with six consecutive histidines, and we experienced poor rates of labeling. However, in some experiments it could be a useful enough tool to justify the inefficiencies.

### SEC purification of polyP

#### Spin columns

We evaluated the use of commercial Zeba spin-desalting columns to separate polyP_1000_ from unbound label after the reaction. After accounting for the reduction in volume after the spin-column purification, about 84% of the polyP added to the column was recovered in the purified material (Table 2). Conversely, 99.4% of the biotin (which was added in large excess) was removed (Table 2), indicating that spin SEC columns can effectively and easily separate polyP_1000_ from the other reaction components.

**Table 2:** Efficiency of spin-column purification of biotinylated polyP_1000_.

	PolyP (as phosphate)			Biotin		
	[P]_initial_ (mM)	[P]_final_ (mM)	% recovered	[Biotin]_initial_ (mM)	[Biotin]_final_ (mM)	% removed
Trial 1	126	93.2	74.0	6.8	0.02	99.68
Trial 2	128.5	103.0	80.2	2.1	0.02	99.05
Trial 3	85.4	82.9	97.1	3.0	0.01	99.54
Mean ± SEM			83.7 ± 6.0	Mean ± SEM		99.42 ± 0.2

Since desalting columns are a size-based separation technique, we evaluated whether these columns could also be used with the much shorter polyP_75_. Accordingly, we added a 4.23 mM polyP_75_ solution to each of four spin columns and evaluated the eluates’ phosphate concentration. We determined that the recovery was 79%, indicating this separation technique is effective for both the longer polyP_1000_ and the shorter polyP_75_ (Table 3).

**Table 3:** Spin column efficiency for PolyP_75_.

	Spin column number				
	1	2	3	4	Mean ± SEM
PolyP concentration in elution (as monophosphate, in mM)	3.45	3.26	3.22	3.42	3.34 ± 0.06
Percent recovery of initial polyP sample loaded	81.6	77.1	76.2	80.9	79.0 ± 1.35

### FPLC

**Fig 5:**
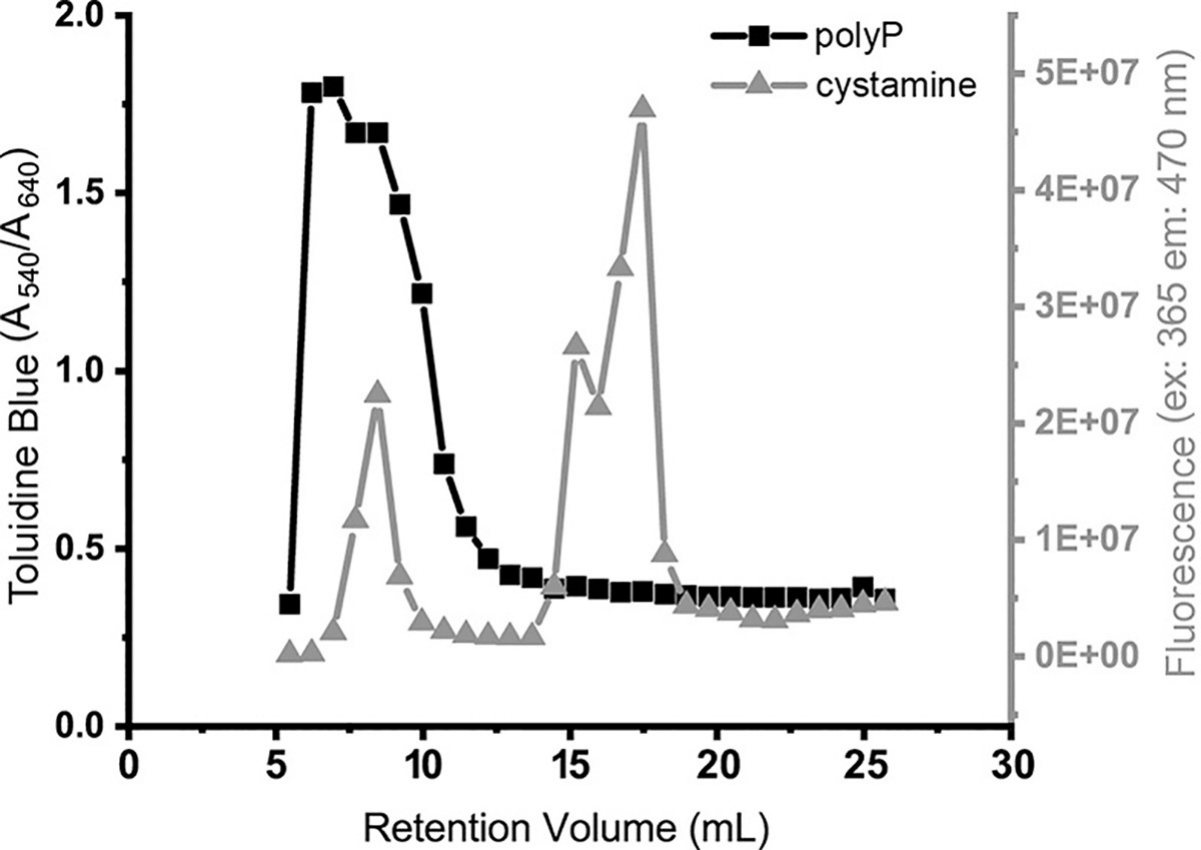
Use of FPLC-based SEC to purify polyP_75_ from free label. PolyP_75_ was reacted with cystamine and EDAC, after which the reaction mixture was chromatographed by SEC on a Superdex 30 Increase 10/300 GL column. PolyP, detected by metachromatic staining with toluidine blue (left *y*-axis), eluted at 6–12 mL. Cystamine, detected by fluorescamine fluorescence (right *y*-axis), eluted in two peaks, corresponding to cystamine covalently bound to polyP (7–10 mL), and free cystamine (15–19 mL).

We also examined the use of higher-resolution, FPLC-based SEC for separating polyP from reactants. A reaction mixture of polyP_75_, EDAC and cystamine was resolved on a Superdex 30 Increase 10/300 GL column (Fig 5). Cystamine-coupled polyP was resolved well from unreacted cystamine.

## Conclusions

We here report the use of various bridging molecules for convenient end-labeling of polyP using a variety of coupling chemistries. For most applications, reacting EDAC with polyP and an appropriate amine at pH 8.0 and 37°C for 1 h will result in efficiently labeled material that preserves the starting polyP polymer length distribution. Using EDAC to covalently couple peptides or other bridging molecules such as cystamine to the terminal phosphates of polyP greatly expands the types of coupling chemistries available for derivatizing polyP, including a variety of commercially available amine-reactive and thiol-reactive labels, as well a click-chemistry partner (DBCO). This allows efficient labeling of polyP of varying polymer lengths with biotin, fluorescent dyes or a peptidyl epitope tag. Previously, EDAC-mediated polyP derivatization was limited to labels with primary amines (or, in special cases, with alcohols), and was problematic if the label contained other reactive groups such as carboxylates (or if the label was not soluble at relatively high concentrations in aqueous solution, since polyP has poor solubility in the presence of organic solvents or cosolvents) [6]. Additionally, we report facile methods for purification of labeled polyP. These methods should facilitate studies of the biological activities of polyP, as well as possible formulation of a variety of materials containing covalently attached polyP. As interest in the biological functions of polyP grows, a wide variety of available modifications could be useful for studies such as western blots, flow cytometry, ELISAs and pull-downs.

## Supporting information

S1 Raw images (PDF)
