# Supplementary material for: Diversification of polyphosphate end-labeling via bridging molecules
Source: PLoS One. 2020 Aug 21;15(8):e0237849. doi: 10.1371/journal.pone.0237849 (PMC7446893; doi:10.1371/journal.pone.0237849)
Supplement: S1 Raw images — (PDF) [file pone.0237849.s001.pdf]

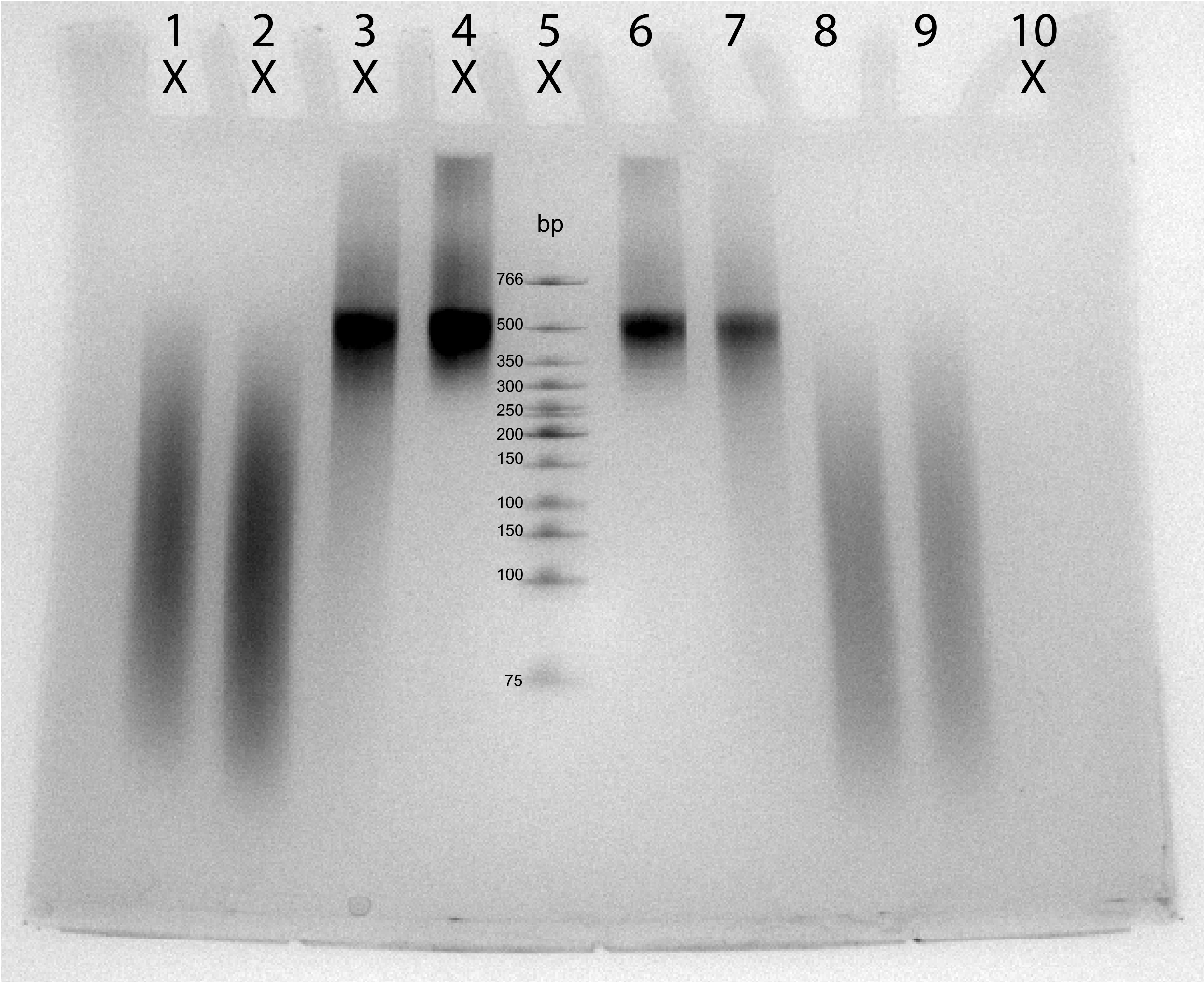

Figure 2A

Mini-Protean gel, 10 well, Bio-Rad 4-20% TBE, stained for polyP using 0.05% toluidine blue in 25% methanol/5% glycerol, followed by destaining in 25% methanol/5% glycerol and imaged using white light

Wells loaded in order:
1. 6 mM $polyP_{narrow}$-biotin reacted at 60ºC 1h (4 µg)
2. 6 mM $polyP_{narrow}$-biotin reacted at 37ºC overnight (4 µg)
3. 6 mM $polyP_{narrow}$-biotin reacted at 37ºC 1h (4 µg)
4. 6 mM $polyP_{narrow}$ starting material (4 µg)
5. Low Molecular Weight DNA Ladder NEB #3233L
6. 3 mM $polyP_{narrow}$ starting material (2 µg)
7. 3 mM $polyP_{narrow}$-biotin reacted at 37ºC 1h (2 µg)
8. 3 mM $polyP_{narrow}$-biotin reacted at 37ºC overnight (2 µg)
9. 3 mM $polyP_{narrow}$-biotin reacted at 60ºC 1h (2 µg)
10. empty

Figure 4B

The same gel (Mini-Protean gel, 15 well, Bio-Rad 4-20% TBE) imaged in two ways

1st (before staining with toluidine blue):
illumination at 302 nm, with a 595 nm emission filter, 45s exposure

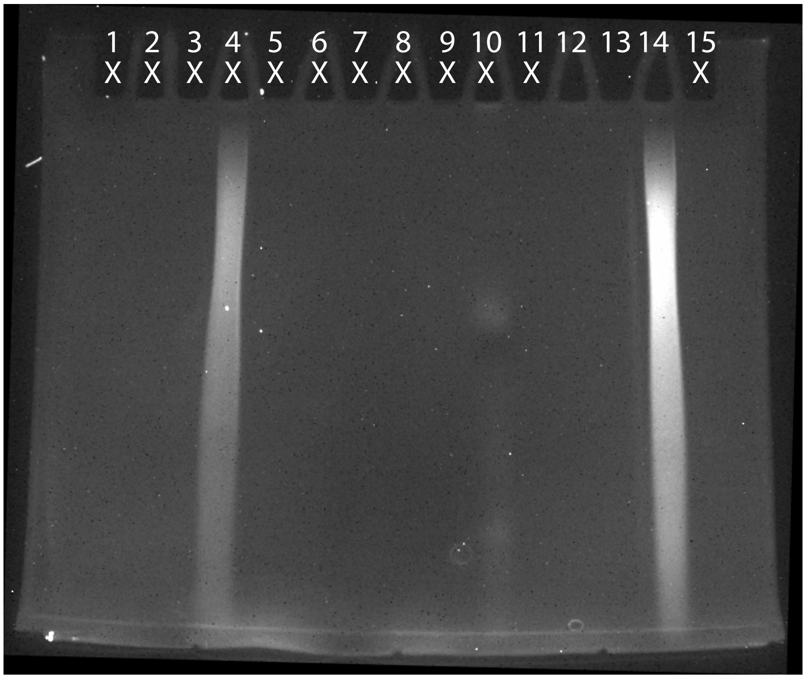

Wells loaded in order:

1. empty
2. 5 mM $polyP_{1000}$ starting material (4 µg)
3. empty
4. 5 mM $polyP_{1000}$-cystamine-DyLight488 (4 µg)
5. empty
6. 1 mM $polyP_{1000}$ starting material (0.8 µg)
7. empty
8. 1 mM $polyP_{1000}$-cystamine-DyLight488 (0.8 µg)
9. empty
10. 50bp DNA ladder NEB# N3236S
11. empty
12. 10 mM $polyP_{1000}$ starting material (8 µg)
13. empty
14. 10 mM $polyP_{1000}$-cystamine-DyLight488 (8 µg)
15. empty

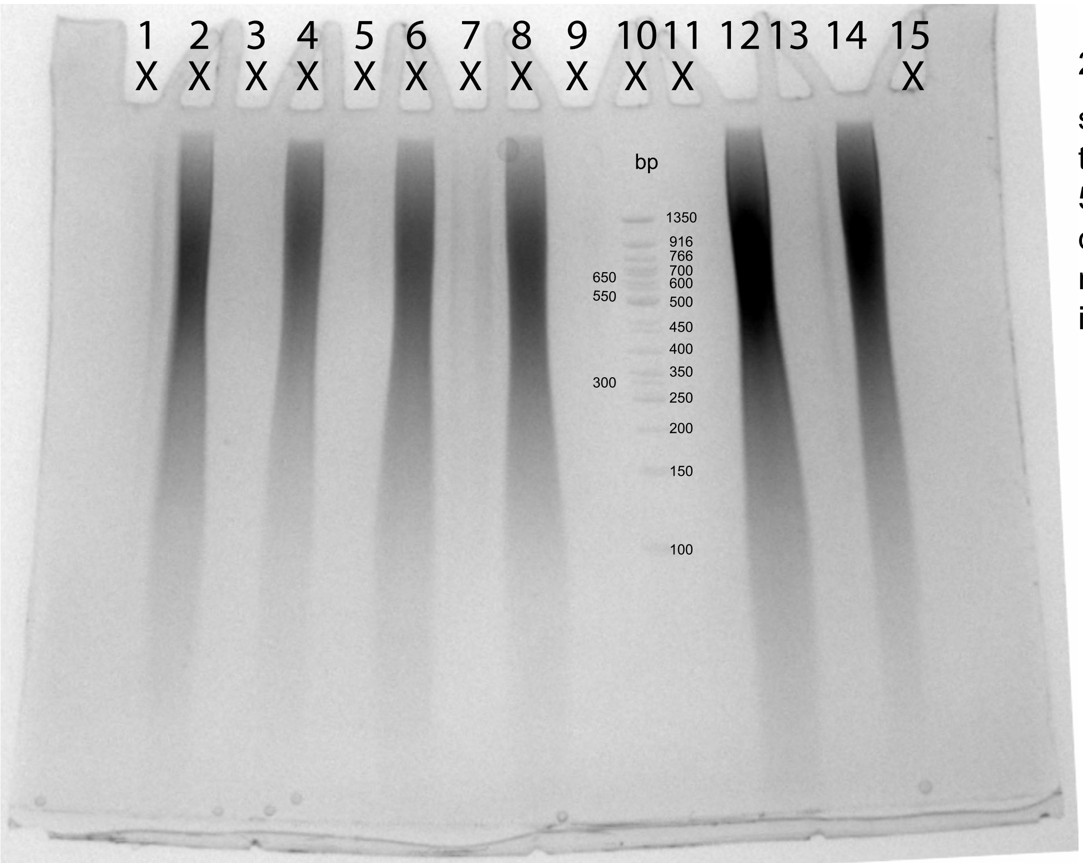

2nd:

stained for polyP using 0.05% toluidine blue in 25% methanol/ 5% glycerol, followed by destaining in 25% methanol/5% glycerol and imaged using white light
